# Case growth analysis to inform local response to COVID-19 epidemic in a diverse U.S community

**DOI:** 10.1038/s41598-022-20502-2

**Published:** 2022-10-04

**Authors:** Marcia C. de Oliveira Otto, Frances A. Brito, Ji Yun Tark, Eric Bakota, Jose-Miguel Yamal, Dritana Marko, Shreela V. Sharma, Michael R. Brown, Savitri N. Appana, Alison M. Rector, Stephen H. Linder, Jennifer Kiger, Karen C. Tseng, Alanna C. Morrison, Eric Boerwinkle

**Affiliations:** 1 School of Public Health, The University of Texas Health Science Center at Houston (UTHealth, 1200 Pressler Street, Suite E-619, Houston, TX 77030-3900 USA; 2 Harris County Public Health, Houston, TX USA; 3 Harris Health System, Houston, TX USA

**Keywords:** Health care, Infectious diseases

## Abstract

Early detection of new outbreak waves is critical for effective and sustained response to the COVID-19 pandemic. We conducted a growth rate analysis using local community and inpatient records from seven hospital systems to characterize distinct phases in SARS-CoV-2 outbreak waves in the Greater Houston area. We determined the transition times from rapid spread of infection in the community to surge in the number of inpatients in local hospitals. We identified 193,237 residents who tested positive for SARS-CoV-2 via molecular testing from April 8, 2020 to June 30, 2021, and 30,031 residents admitted within local healthcare institutions with a positive SARS-CoV-2 test, including emergency cases. We detected two distinct COVID-19 waves: May 12, 2020–September 6, 2020 and September 27, 2020–May 15, 2021; each encompassed four growth phases: lagging, exponential/rapid growth, deceleration, and stationary/linear. Our findings showed that, during early stages of the pandemic, the surge in the number of daily cases in the community preceded that of inpatients admitted to local hospitals by 12–36 days. Rapid decline in hospitalized cases was an early indicator of transition to deceleration in the community. Our real-time analysis informed local pandemic response in one of the largest U.S. metropolitan areas, providing an operationalized framework to support robust real-world surveillance for outbreak preparedness.

## Introduction

The ongoing coronavirus disease 2019 (COVID-19) pandemic continues to impose substantial burdens on United States (U.S.) communities and the health systems caring for them. As of February 2022, at least 24% of U.S. residents had been infected by severe acute respiratory syndrome coronavirus 2 (SARS-CoV-2), the pathogen that causes COVID-19, and more than one in every 500 Americans had died of COVID-19^1^. During the first large U.S. outbreak wave in the summer of 2020, nearly 30% of individuals infected with SARS-CoV-2 required hospitalization^2^, and 15–27% of admitted patients required intensive care unit (ICU) admission or invasive mechanical ventilation^2, 3^. The immense scale, dynamic nature, and severity of the pandemic warranted both swift and sustained response efforts to mitigate transmission as well as to inform healthcare needs and preparedness.

One of the major barriers to pandemic response has been the lack of reliable, objective detection of new outbreak waves and its potential impact on severe disease and hospitalization in real time. While numerous forecast models relying predominantly on machine learning techniques to analyze national, statewide, or a combination of various data sources are currently available^4, 5^, substantial uncertainty surrounding model predictions impedes informed decisions about the likelihood, timing, or magnitude of upcoming waves^5^. Recently, Utsunomiya and colleagues developed a new framework to identify outbreak waves using real-time worldwide data from the European Center for Disease Prevention and Control (ECDC)^6^. Unlike machine learning models, Utsunomiya’s approach utilizes moving regression technique and Hidden Markov models to systematically identify four distinct growth stages: lagging (i.e. beginning of the outbreak), exponential or rapid growth, deceleration, and stationary (i.e. near zero growth). This novel analysis produces more accurate prediction of new cases; however it focused primarily on national-level estimates and not on city or county-level data, which most closely reflect the changes in SARS-CoV-2 transmissibility in local communities and, thus, are critical to guide local public health response. In addition, most studies evaluating case dynamics to date^6–12^ focused on community-based data or on hospital system records, but not both. Community-based data reflect the spectrum of infection from asymptomatic to severe cases, while inpatient hospital data include more severe cases requiring hospital care^13^. Crucial to effective resource planning, the time period between a new outbreak is detected in the community, and increased rapid rise in the number of SARS-CoV-2 infections among hospitalized individuals remains mostly unknown. The use of COVID-19-related hospitalizations in the community has been recently highlighted by the Centers for Disease Control and Prevention as an important metric reflecting the potential for strain on the local health system and COVID-19 cases in the community^14^. Such knowledge is crucial to inform decisions about community prevention strategies and to enable hospital systems to prepare for, and manage, potential surges in the demand for hospital beds, ICU beds, and ventilators. Finally, most studies^6–12^ have focused on a single major outbreak wave, providing little insight into how SARS-CoV-2 in the community and among patients in hospital settings may have changed throughout the pandemic.

To address these key gaps in knowledge, we used community case investigation and hospital data from one of the largest U.S. metropolitan areas to systematically characterize distinct phases of two major SARS-CoV-2 outbreak waves in Harris County, and determined the transition times from rapid spread among community residents to increased rates of infected patients hospitalized in local healthcare systems.

## Materials and Methods

### Study setting and population

Our analysis included individuals testing positive for SARS-CoV-2 from April 8, 2020 to June 30, 2021 using de-identified individual-level SARS-CoV-2 molecular testing data from both the Harris County Public Health (HCPH) Department and the Texas Medical Center (TMC), a healthcare system comprising seven hospitals and outpatient facilities located in the Greater Houston area. Community data from Harris County Public Health Department jurisdiction included confirmed SARS-CoV-2 infections among the 2.4 M Harris county residents who live outside the service area of the City of Houston. The second dataset included patients admitted within TMC institutions with a positive SARS-CoV-2 test, including emergency cases. Throughout the course of the pandemic, new datasets were shared with UTHealth every week. UTHealth subsequently would present reports and analysis during regular weekly meetings with TMC and Harris County leadership. We used ZIP code information to restrict our analysis to TMC inpatients residing within Harris County community. Self-reported demographic information was obtained during case investigation, scheduling, registration of the test at designated sites, or from patient records. Information on daily tests in Harris County was obtained from the HCPH Laboratory database. All methods were carried out in accordance with reporting guidelines and regulations. Informed consent from all participants in HCPH data was obtained at testing sites. Informed consent was not sought for the TMC cohort study since it used anonymous data and was not designated as human subjects research by the Western Institutional Review Board. The study protocol was reviewed and approved by the committee for the protection of human subjects of the University of Texas Health Science Center at Houston.

### Identifying growth phases

We adapted the framework of Utsunomiya et al.^6^ for identifying county-level SARS-CoV-2 phases of each wave in community and hospital settings. This method begins by estimating the speed in which the number of infections is increasing, or case growth rate (cases/day), and the rate of change in the daily cases, or case growth acceleration (cases/day^2^). We used a moving regression, whereby we fit a regression line to the cumulative case curve over a given window of time, using the slope of the line to estimate the growth rate at that time point, and repeating this process by shifting the window of time by one day. After obtaining estimates of daily growth rates, we repeated the moving regression process for case growth acceleration over time. The moving regression step included an adjustable smoothing parameter, *s*, that determined the window of time, *k* = 1 + 2* s* days, to which the regression curve is fit. An *s* value of 5, or an 11 day window, provided a good fit to the data by sufficiently reducing noise due to weekly fluctuations in case reporting. The growth acceleration estimate was then used to fit a Hidden Markov model for growth stage classification. We followed Utsunomiya et al.’s outline for the classification step, including the same initial, emission, and transition probability matrices to identify four distinct growth phases: (1) lagging, (2) exponential or rapid growth, (3) deceleration, and (4) stationary/linear^6^. Lagging was defined as the introduction of SARS-CoV-2, when there is a low number of new daily cases. An exponential phase was defined as the period of rapid increase in the number of new daily cases. A deceleration phase occurred when there was a sharp decline in new daily cases, typically following an exponential phase. Lastly, a stationary/linear phase marks a time when the number of new daily cases remains mostly stable or continues to change at lower rates. Here, we combined both stabilization and linear growth periods proposed by Utsunomiya et al. for simplicity and ease of interpretation.

**Figure 1:**
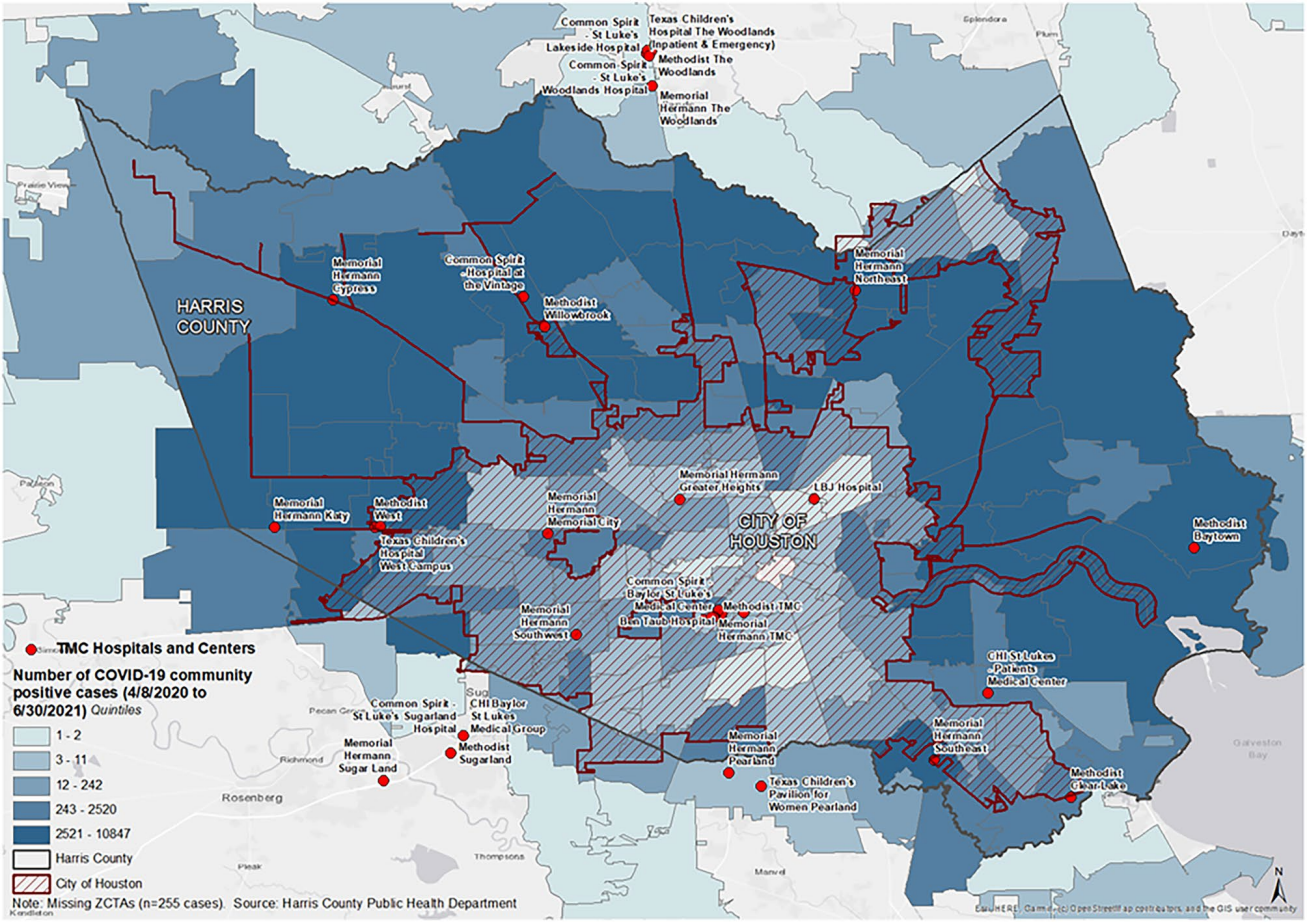
Distribution of cumulative SARS-CoV-2 infections across ZIP Code Tabulation Areas in Harris County, Texas, from April 8, 2020 to June 30, 2021. All SARS-CoV-2 infections were summed across ZIP Codes and then across US Census ZIP Code Tabulation Areas (ZCTAs) based on the individuals’ address information. Red circles indicate the location of Texas Medical Center (TMC) facilities. Geospatial distribution patterns vary from light to dark blue according to increasing quintiles of cumulative infections during the analysis period. 255 records were excluded due to missing ZIP Code information. Figure was generated using ArcMap (ArcGIS version 10.8.2).

The classification step requires a selection of an acceleration cutoff (*c*), a free parameter, that helps determine shifts between phases. A larger acceleration cutoff leads to the least number of phase transitions while a smaller acceleration cutoff leads to flexible phase transitions. Further, acceleration cutoffs are sensitive to small population sizes due to a smaller acceleration (cases/day^2^) range. We varied *c* between 1 and 10 and found that *c* = *3* and *c* = *1* for the community and TMC, respectively, provided sufficient classification results by not being overly sensitive to localized changes in acceleration while also detecting transitions in phase dynamics. The beginning of a wave was identified as the first date of the first observed exponential phase. The end of a wave was determined by the last date of the last deceleration phase and before entering a sustained linear phase. We compared transition times, peaks, and duration of outbreak waves among Harris County community residents and TMC inpatients. We estimated daily growth rate adjusted for population size (i.e., daily cases per 100,000 residents) to evaluate the risk of exposure to SARS-CoV-2 across age groups. We conducted comparisons of growth rates between community and inpatient groups, overall and by age groups, for each outbreak wave. To explore a metric capturing severity of COVID-19 in the region and the relative impact of community infections on hospital admissions in Harris County, we estimated inpatient-community case ratio. This measure is comparable to infection-hospitalization ratio^15^, however its numerator is restricted to patients admitted within TMC institutions. Average and interquintile range of daily tests administered per 100,000 residents were calculated overall, and for each outbreak wave, reflecting testing coverage during the analysis period. All analyses were performed using R version 4.0.2. Figure 1 generated using ArcMap (ArcGIS version 10.8.2 https://www.esri.com/en-us/arcgis/products/arcgis-desktop/resources ).

## Results and Discussion

In this study, we used local SARS-CoV-2 community and hospital data from a large, diverse U.S. metropolitan area to detect new outbreak waves among local residents, characterize specific wave periods among community residents (i.e. lagging/rapid growth, deceleration and stationary), and quantify the time period from the emergence of a new outbreak wave among local residents to accelerated rates of admission of infected residents in local healthcare systems. Our analysis detected two large waves of SARS-CoV-2 in Harris County between April 8, 2020 and June 30, 2021, leading to a total of 193,237 laboratory-confirmed cases among individuals living in the Harris County jurisdiction area, and 30,031 hospitalized residents (Supplemental Table 1). The emergence of two large waves during the analysis period is consistent with infection trends observed in Texas, the U.S. and worldwide^1, 16^, confirming the cyclical nature of SARS-CoV-2 transmission overtime^17^.

Figure 1 shows a map of Harris County where local SARS-CoV-2 cases were identified during the analysis period, as well as local hospitals contributing to this analysis. Compared to overall community residents, hospitalized cases were more likely to be older [31.7% inpatient cases ≥ 65 years (y) versus 9.8% community cases; Supplemental Table 1]. When comparing racial/ethnic distributions between community and hospital cohorts, inpatient cases had a higher proportion of individuals self-identified as Hispanic, White, or Black individuals; however, 37% of community cases did not have a reported race or ethnicity (Supplemental Table 1). During the analysis period, the average (interquintile range, IQR) number of daily tests per 100,000 residents in Harris County was 91 (12, 160). The average number of daily tests per population increased substantially across waves [the average (IQR) number of daily tests per 100,000 residents was 24 (7, 38) and 141 (24, 247) for the first and second waves, respectively].

### Wave 1: May 12, 2020–September 6, 2020

**Figure 2:**
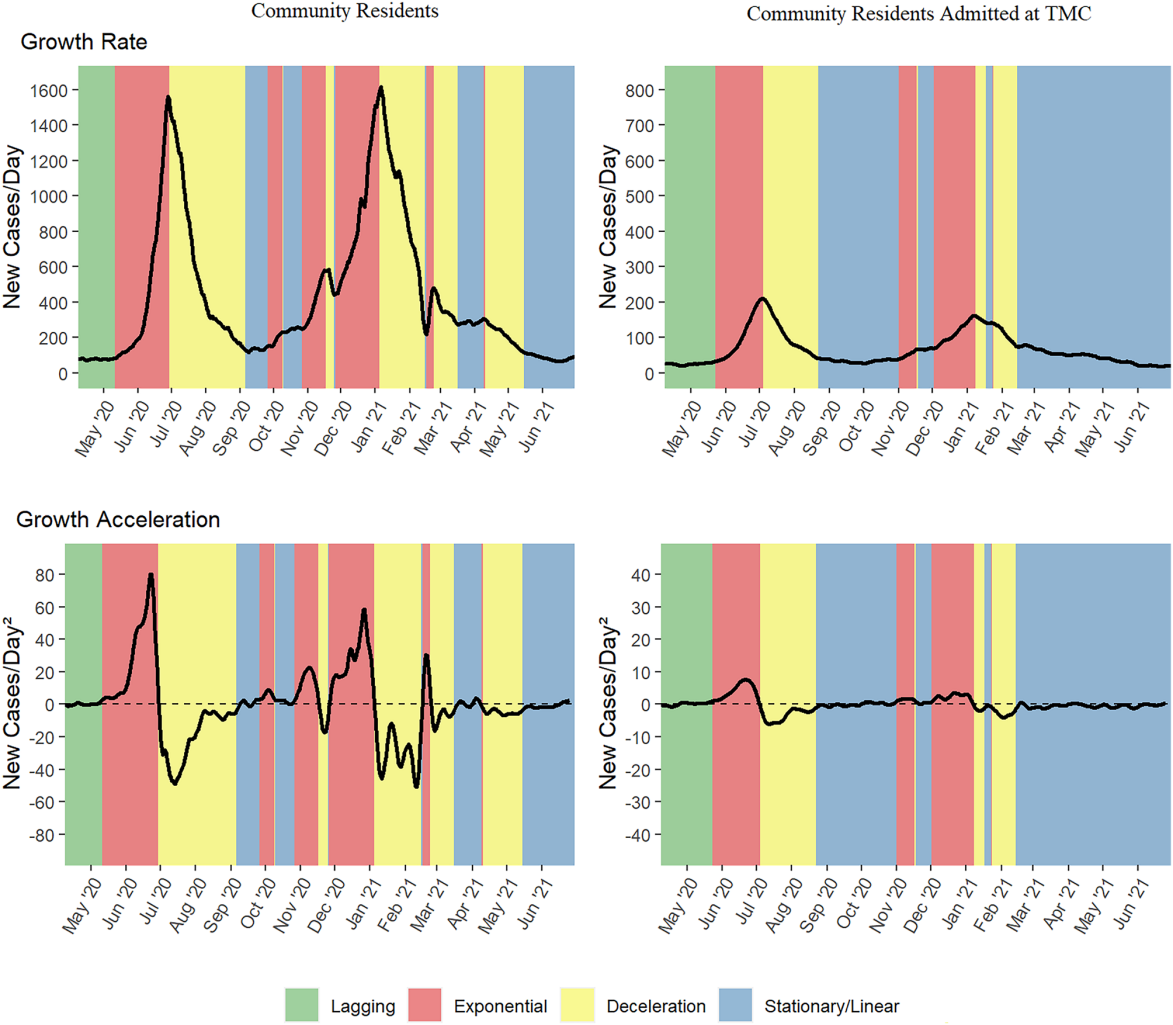
SARS-CoV-2 growth rate, acceleration and stage among Harris County residents (left) and a subgroup of Harris County residents admitted to Texas Medical Center(TMC) hospitals with a positive SARS-CoV-2 test (right) from April 8, 2020 to June 30, 2021.

The first large SARS-CoV-2 outbreak wave in the Harris County community data began with transition from lagging to exponential growth phase on May 12, 2020. After 49 days of rapid case rise in the community, the county reached its maximum growth rate (~ 1,500 cases per day), transitioning to a deceleration stage starting June 30, 2020. The number of daily cases decreased rapidly for 69 days, reaching a linear/stabilization stage on September 7, 2020 (Figs. 2 and 4). In total, 63,026 cases were detected in the community over a period of 118 days of exponential growth and rapid deceleration. The median age (IQR) for community cases was 38 (25, 52) y. Population-adjusted growth rates by age groups revealed the highest rates among adults 20–39 y, followed by adults 40–64 y and 65 + y; the number of daily cases was lowest among children (< 10 y) and adolescents (10–19 y) (Table 1).

**Table 1:** Daily growth rates* in SARS-CoV-2 cases per 100,000 residents during outbreak waves in Harris County, TX, stratified by age group.

	Overall (April 8, 2020 – June 30, 2021)			May 12, 2020 – September 6, 2020			September 27, 2020 – May 15, 2021		
	Average daily cases per 100,000 residents*			Average daily cases per 100,000 residents			Average daily cases per 100,000 residents		
	Community^a^ (N = 193,237)	Inpatients^b^ (N = 30,031)	Cumulative inpatient: community case ratio ^c^	Community^a^ (N = 63,026)	Inpatients^b^ (N = 10,494)	Cumulative inpatient: community case ratio	Community^a^ (N = 120,855)	Inpatients^b^ (N = 16,931)	Cumulative inpatient:community case ratio
**Age (years)**									
< 10	5.0	1.0	0.17	4.5	1.-5	0.31	8.1	0.8	0.10
10–19	12.1	0.9	0.06	12.1	1.1	0.09	18.8	0.7	0.04
20–39	24.8	2.2	0.09	33.4	2.6	0.08	28.4	1.9	0.07
40–64	20.9	4.6	0.22	26.2	4.9	0.19	24.7	4.0	0.16
65 +	16.9	11.4	0.67	21.4	10.3	0.49	20.0	10.0	0.50
All	18.0	1.0	0.05	5.9	1.0	0.17	21.8	3.0	0.14

* New cases/day in 100,000 population; ^a^ SARS-CoV-2 confirmed cases in Harris County community; cases include all local SARS-CoV-2 infections; ^b^ Harris County community residents admitted within TMC hospitals with a positive SARS-CoV-2 test. ^c^The inpatient community case ratio was calculated as the number of Harris County residents who were admitted within TMC institutions divided by the total number of cases among residents at the same time period.

Among hospitalized cases, transition to rapid growth in the number of cases was detected on May 24, 2020. The exponential phase among hospitalized residents lasted 42 days, reaching a peak growth rate of nearly 210 cases/day. The rapid growth phase was followed by a deceleration period from July 5, 2020 until August 23, 2020, which marked the beginning of a 71-day stationary period. In total, 10,494 inpatient cases were identified during critical stages of SARS-CoV-2 outbreak. The median age for inpatient cases was 55 (38, 68) y. Compared to community cases, hospitalized residents were more likely to be middle-aged or older adults (41% inpatient cases 40–64 y vs 37% community cases; 29% inpatient cases 65+ y vs 10% community cases, Supplemental Table 2).

### Wave 2: September 27, 2020–May 15, 2021

Transition to an exponential growth phase in the second large wave in Harris County occurred after 20 days of stabilization, on September 27, 2020. Unlike the previous outbreak wave, the second large wave was characterized by mixed growth patterns, with short periods of transition within exponential and deceleration phases (Fig. 2). For example, our analysis detected an 8-day deceleration period during the week preceding the U.S. Thanksgiving holiday on November 25, 2020. Change to a non-transient deceleration stage started January 6, 2021 after 101 days of mixed growth patterns (Fig. 2). During the downward phase, our analysis detected a 7-day rapid growth phase following Winter Storm Uri, a snow and ice storm occurring during February 13–17, 2021 and exerting major impacts across the U.S., Northern Mexico, and parts of Canada. The rapid decline in the number of new cases was interrupted again by a 24-day stationary phase from March 17, 2021 to April 9, 2021, followed by a new deceleration period ending May 16, 2021. A mixed pattern of growth, deceleration, and stationary stages is consistent with findings from case growth analysis by Utsunomiya and colleagues^6^ using national-level data from South Korea, Austria, and China, and could be partially explained by disruptions in data collection and processing, as well as in testing patterns. The maximum growth rate was ~ 1,650 cases/day. The median age (IQR) for cases among community residents was 37 (23, 52) y.

Rapid increase in hospitalized cases was detected starting November 2, 2020, 36 days after detection of the second major wave in Harris County. Similar to patterns in the community, we observed mixed growth patterns among hospitalized cases, with short periods of stabilization that included the Thanksgiving holiday (Fig. 2). This may partially reflect changes in hospitalization patterns during a major national holiday. Deceleration began on January 9, 2021 after 68 days of predominantly upward growth in hospitalized cases. This phase ended February 14, 2021; however, the number of daily inpatient cases continued to decrease at a slower, linear rate for 136 days. The maximum growth rate was ~ 180 cases/day. In total, 16,931 individuals were admitted to TMC hospital and clinics with confirmed SARS-CoV-2 infections. The median (IQR) age for inpatient cases was 58 (42, 70) y. Consistent with patterns observed during the previous wave, hospitalized cases were more likely to be middle-aged or older adults (41% inpatient cases 40–64 y vs 36% community cases; 34% inpatient cases 65 + y vs 10% community cases, Supplemental Table 2).

### Time lag between critical stages of the pandemic in the community and inpatients in local hospital system

**Figure 3:**
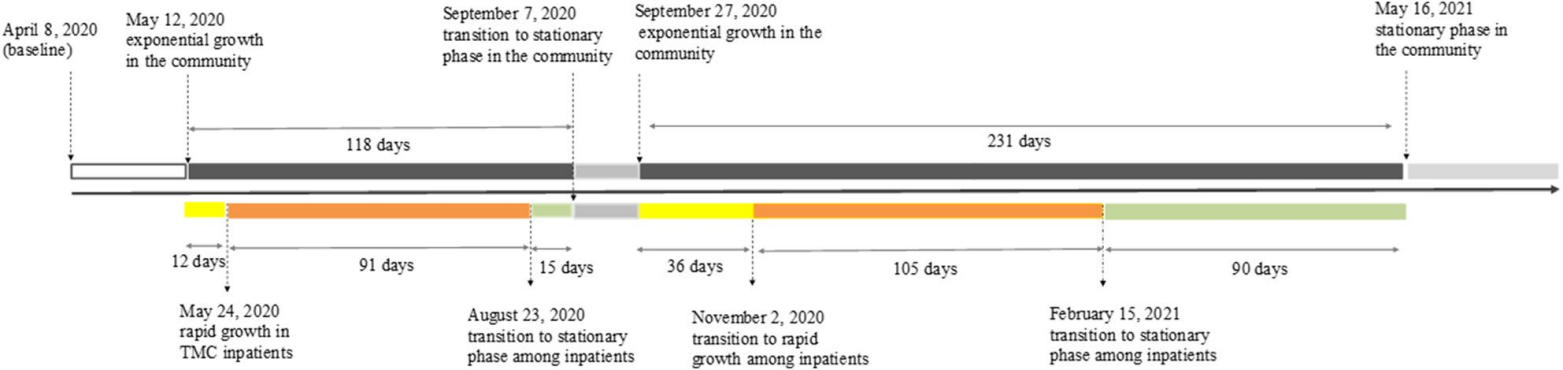
Transition periods to SARS-CoV-2 outbreak in Harris County community and among inpatients in Texas Medical Center. Critical phases of SARS-CoV-2 outbreak (i.e., exponential growth + deceleration stages) in Harris County community (top row) and among TMC inpatients (bottom row). Dark gray areas indicate exponential growth and deceleration stages. Yellow areas indicate lag periods between transition to exponential growth in the community and in local hospitals. Light green areas indicate lag periods between transition to stationary/linear growth in the community and in local hospitals.

To estimate the time period from rapid growth in community transmission to increased rates of COVID-19 related hospitalizations, we compared transition times of outbreak waves among local residents to those of a subgroup of individuals admitted to local healthcare systems with a confirmed SARS-CoV-2 infection. According to the Centers for Disease Control and Prevention (CDC), the number of new COVID-19 related admissions in a given population serves as a proxy for underlying disease severity among community residents and for the ability of local healthcare systems to support additional people requiring hospital care^13^. Figure 3 shows the lag between critical stages of the pandemic in the community and among TMC hospitalized patients. During the first large outbreak wave, a rapid increase occurred in the number of hospitalized individuals with SARS-CoV-2 infections, including admissions for COVID-19, 12 days after the initial stages of the outbreak in the community (i.e., the start of rapid growth stage). On the other hand, early signs of stabilization in infection rates were first observed among TMC inpatients, 15 days before case stabilization in the community. During the second large wave, the lag between rapid community transmission and exponential growth among inpatients was more than two times that of the first large wave (36 days). Consistent with patterns observed in the first wave, transition to stationary/linear period occurred first among hospitalized individuals, with a 90-day lag period until stabilization of infection rates in the community.

### SARS-CoV-2 infections and related hospital admissions by age groups

**Figure 4:**
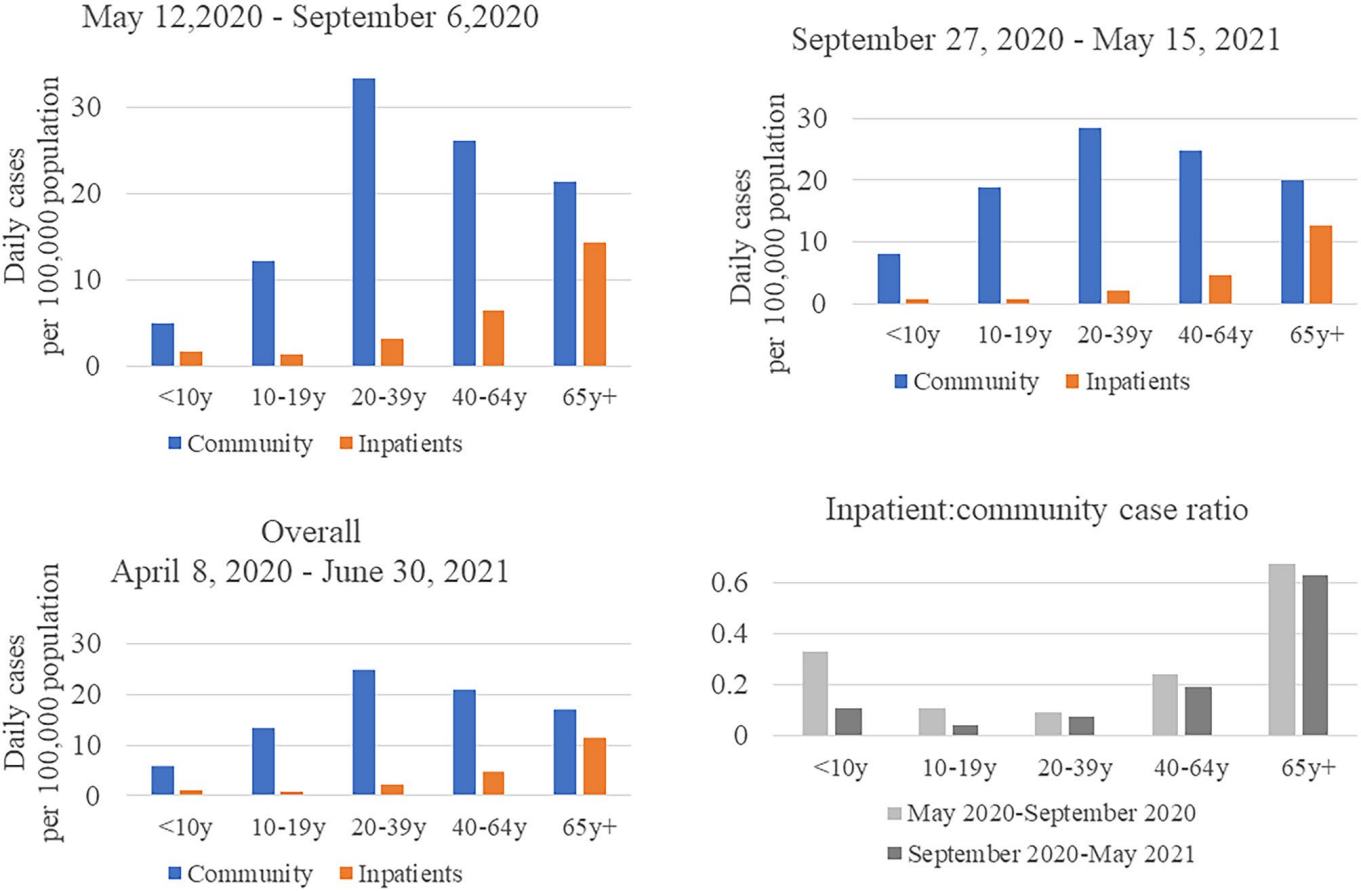
Daily growth rate in SARS-CoV-2 cases per 100,000 population among community residents^a^ and inpatients^b^ during outbreak waves in Harris County, stratified by age group. (**A**) May 2020 – September 2020, (**B**) September 2020 – May 2021, (**C**) Overall Period (April 2020 – June 2021), (**D**) Inpatient:community case ratio. ^a^Community cases include all residents of Harris County with confirmed SARS-CoV-2 infections; ^b^ Inpatients include Harris County residents admitted to TMC hospitals with a positive SARS-CoV-2 test.

Among community cases, the average (IQR) daily growth rate during 448 days was 18 (5, 29) daily cases per 100,000 residents. Population-adjusted growth rates by age groups indicated the highest average growth rates during both waves were among adults 20–39 y, followed by middle-aged adults (40–64 y), and older adults (65 + y) (Table 1; Fig. 4); the average number of daily cases was lowest among children (< 10 y) and adolescents (10–19 y). Similar growth patterns by age group occurred during the first and second waves, suggesting that risk of SARS-CoV-2 infections in the community during the analysis period was highest among adult subgroups, particularly among young and middle-aged adults. These findings align with regional patterns in the southern U.S. during June–August 2020^9, 18^; however, estimates from previous studies were limited to a narrow time window. Among hospitalized cases, population-adjusted rates were the highest rate among older adults, followed by middle aged, and young adults. When we compared inpatient-community case ratios, a metric used as a proxy for severity of COVID-19, we observed a trend toward higher estimates across age groups. Consistent with previous reports ^2, 9, 19, 20^, our analysis confirms that risk of severe illness leading to hospitalization was higher among older residents in Harris County during early stages of the pandemic.

### Sensitivity analyses

In order to evaluate the potential impact of limited testing on the detection of new outbreak waves, we expanded the analysis period to start in January 24, 2020, when results from the first tests where available in the database. This sensitivity analysis detected a short outbreak wave in Harris County between March 14, 2020 and March 31,2020, leading to 850 SARS-CoV-2 cases among residents and 340 hospital admissions (peak growth rate 86 and 34 cases per day among community residents and admitted cases in the community, respectively). Characterized by 17 days of rapid growth, 1 day of deceleration and 41 days of post-wave stabilization, this initial outbreak was detected simultaneously in the community and local hospitals. This is likely to reflect suboptimal surveillance early in the pandemic, when testing was mostly unavailable outside large healthcare systems. In addition, there were no guidelines or protocols in place for testing asymptomatic individuals, potentially delaying the detection of a new infection outbreak in the community until symptomatic residents began to be admitted in local hospitals. Notably, testing uptake increased substantially in Harris County during early stages of the pandemic, with 7 day moving average of daily tests increasing from nearly 3 daily tests during the first week of January 2020 to ~ 200 daily tests during the first week of March 2020. While low testing uptake delayed detection of a new outbreak among community residents, our analysis showed sensitivity to detect an initial outbreak in a large metropolitan region with testing as low as 11 daily tests per 100,000 residents.

To better understand how the emergence of new variants and changes in immunity from vaccination or prior infections may impact the lag time between transition to accelerated spread of infection and rapid rise in hospital admissions, we expanded the analysis to include the most recent time period, following the introduction of COVID-19 vaccines, and the emergence of the delta and omicron as variants of concern^21^. Our analysis detected the transition to the ‘Delta’ wave in Harris County starting June 29, 2021, when 28% of eligible Harris County residents (i.e. residents 12y and older) where fully vaccinated. Exponential growth among hospitalized patients was detected nine days later on July 8 2021. A subsequent outbreak in the community, the ‘Omicron’ wave, was identified following Thanksgiving holiday on November 26,2021, when 45% of eligible residents were fully vaccinated, followed by accelerated growth among hospitalized patients 10 days later on December 6, 2021. Start and end dates for Delta and Omicron waves among Harris County community residents [admitted patients] were Jun 29, 2021 through October 27, 2021 [July 8, 2021 through August 17,2021], and November 26,2021 through March 10,2022 (December 6, 2021 through March 6, 2022). Overall, our findings confirmed reports of higher transmissibility and relatively lower hospitalization among community residents (see Supplemental Fig. 1). Notably, lag periods and temporal patterns are consistent with those observed early in the pandemic. Recognizing the complexity and fast changing nature of risk factors for COVID-19- related hospitalization at the population level including virulence, population immunity from vaccination and prior SARS-CoV-2 infections, prevalence of comorbidities, access to health care and treatment, which are unavailable or poorly captured in case investigation and hospital data, our analysis did not aim to predict new outbreak waves or the lag period until rapid increase in hospitalization. Rather, we worked toward strengthening local disease surveillance by providing objective identification of new outbreak waves, quantifying the magnitude of community transmission and related hospitalization in real time, guiding local response during critical periods of the COVID-19 pandemic.

This study relied on real-world, county-level and hospital system-based data in one of the largest U.S. metropolitan areas. We characterized distinct phases of two major SARS-CoV-2 outbreak waves, and determined the transition times from critical outbreak phases among community residents to sharp increase in cases hospitalized in local healthcare systems. Our analysis confirmed that during early stages of the COVID-19 outbreak with broad availability of testing in the community and hospital systems, the surge in the number of daily cases in the community preceded that of local hospitals by 12 to 36 days, while rapid decline in the number of hospitalized cases was an early indicator of transition to deceleration in SARS-CoV-2 transmission. Our work builds on and extends the literature by leveraging real-world data derived from both public health department and hospital records, providing a real-time detection of outbreak phases among community residents as well as among individuals hospitalized within a local health system, who are more likely to be critically ill. Conducted on a continuous basis with weekly refreshes of data, our analyses helped inform the local response during early stages of the pandemic. Presented during weekly briefings with public health leadership, healthcare CEOs and school superintendents, our case growth analysis was relied upon as their early warning system. Decisions on resource needs and control measures needed to be anticipatory, as infection rates formed successive waves over time which repeatedly stressed the public health system.

**Figure 5:**
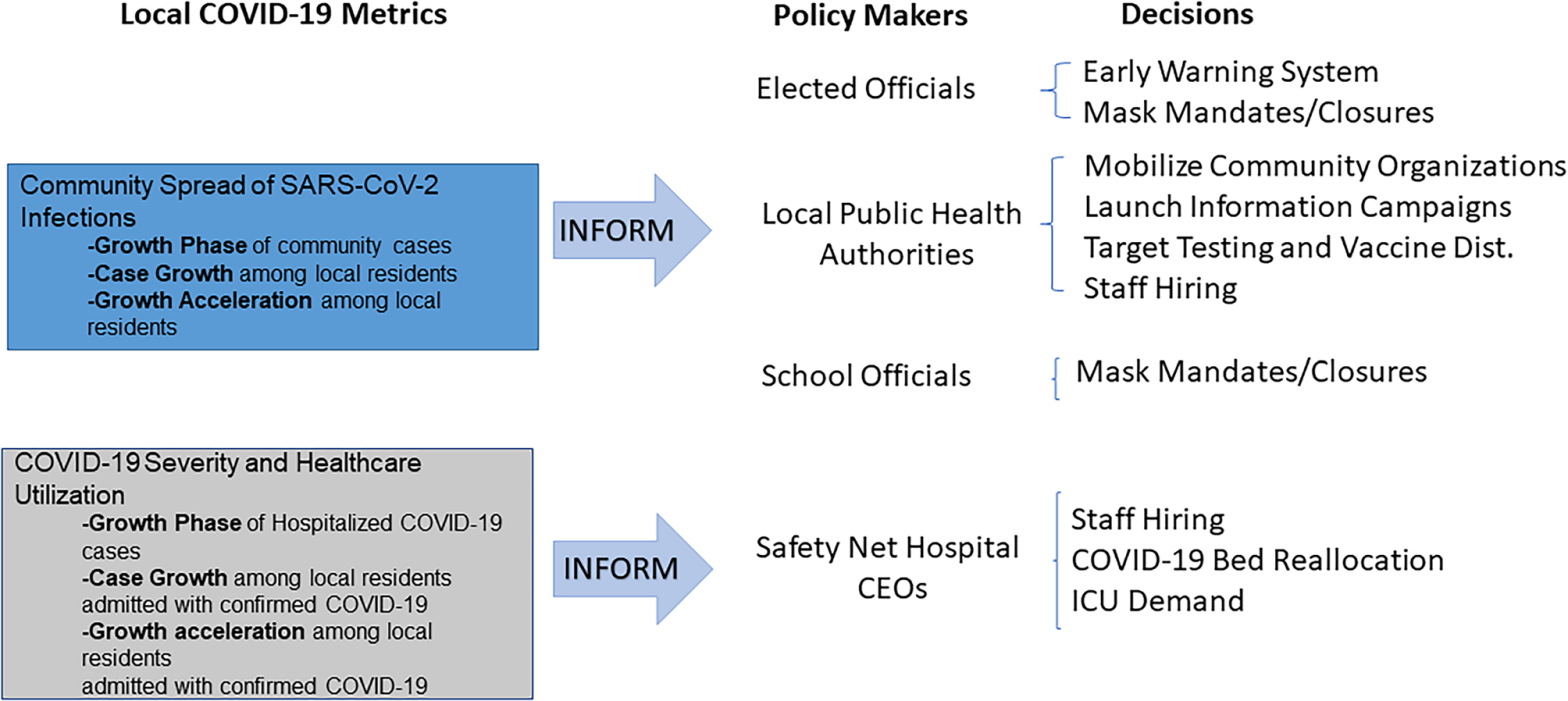
Community metrics informing response to COVID-19 pandemic.

Accordingly, one key accomplishment of this work was procedural: we built a 4-party collaborative process, extending well beyond the provision of fresh indicators on a weekly basis, to incorporate an *exchange* relationship that cut across jurisdictions and levels of authority. Each meeting raised issues of practical implications and proper interpretation. Each party faced their own set of challenges and public responsibilities. Questions (and frustrations) were brought forward, and expectations were set and reset, reshaping our interactions over time. The four policymaking parties, along with examples of the decisions they faced, are listed in Fig. 5. All four represent tax-payer funded entities; three of the four have explicit community health mandates. All were pressed to make decisions based on partial and rapidly changing information. Case growth analyses informed these decisions and became a standard feature and highlight of our briefings.

A key innovation of this work is the operationalization of data analysis methods that enable robust, real-time characterization of transmission dynamics to inform both surveillance approaches and healthcare preparedness. By analyzing case dynamics over a 15 month period, we were able to characterize outbreak waves during periods of major changes to factors influencing the intensity and duration of a local outbreak, including environmental factors (e.g., seasonal changes in temperature and humidity), behavioral and policy factors (mask use requirements, measures to restrict mobility, school and business closing, stay at home orders), and the introduction of new COVID-19 treatments, vaccines, and SARS-CoV-2 pathogen variants. Consistent with growth rate trends observed in Texas, the U.S. and worldwide^1, 16^, the emergence of two large waves during the initial 15-month period supports the hypothesis that peaks of SARS-CoV-2 transmission could follow a seasonal pattern^17, 22, 23^. The longer duration of the second wave and its higher peak growth rates suggest higher SARS-CoV-2 transmissibility under cold and dry winter conditions as previously reported^17^. At the same time, lower average acceleration and slower case growth among hospitalized cases during the second wave outbreak could be partially influenced by improvements to treatment protocols and increasing immunity to SARS-CoV-2 due to previous infections and vaccination coverage, particularly among older adult groups at higher risk of severe disease and hospitalization.

Findings from our analysis may not be replicated over time as conditions surrounding SARS-CoV-2 transmission and related hospitalization continue to change. Given the complex and dynamic nature of determinants of community transmission, it will be crucial to use real-time analysis to continuously inform public health response to future outbreaks.

Our study has potential limitations. Access to testing was limited during early stages of the pandemic, which may have affected case estimates during this period. Testing in the community was often limited to individuals experiencing symptoms or those with concern about exposure to SARS-CoV-2, which may have underestimated true growth rates. Also, hospitalized cases include individuals hospitalized due to COVID-19, as well as individuals who tested positive for SARS-CoV-2 while hospitalized for non-COVID-19–related procedures.

## Conclusion

The COVID-19 pandemic has had a major impact on global health. Our findings confirmed that during early stages of the COVID-19 outbreak in Harris County, Texas, the surge in the number of daily cases in the community preceded that of local hospitals by 12–36 days, while rapid decline in the number of hospitalized cases was an early indicator of transition to slower community transmission. To our knowledge, this is the first study to operationalize methods to monitor case growth in real time using county-level data and hospitalization records. Our analyses helped inform public health response during early stages of the pandemic, providing an important platform to strengthen local surveillance to monitor and respond to future outbreaks.

## Supplementary Information

Supplementary Information.

## Data Availability

The data supporting the findings of this study are available at https://covid-harriscounty.hub.arcgis.com/ and https://www.tmc.edu/coronavirus-updates/.
