# Supplementary material for: Case growth analysis to inform local response to COVID-19 epidemic in a diverse U.S community
Source: Sci Rep. 2022 Oct 4;12:16217. doi: 10.1038/s41598-022-20502-2 (PMC9532394; doi:10.1038/s41598-022-20502-2)

Supplemental Table 1. Demographic characteristics of individuals testing positive for SARS-CoV-2 in Harris County and Texas Medical Center between April 8, 2020 and June 30, 2021

	**Community Residents[a] (N=193,237)**	**Inpatients[b] (N=30,031)**
**Age (years)**		
<10	9,140 (4.7%)	1,398 (4.7%)
10–19	23,508 (12.2%)	1,269 (4.2%)
20–39	71,795 (37.2%)	5,436 (18.1%)
40–64	69,753 (36.1%)	12,414 (41.3%)
65+	18,932 (9.8%)	9,514 (31.7%)
**Sex**		
Female	96,782 (50.1%)	1,814 (6.0%)
Male	84,571 (43.2%)	1,895 (6.3%)
Missing/Unknown	12,884 (6.7%)	26,322 (87.6%)
**Race/Ethnicity[c]**		
Hispanic or Latinos	70,701 (36.6%)	12,023 (40.0%)
White	27,923 (14.5%)	5,821 (19.4%)
Black	16,903 (8.7%)	5,280 (17.6%)
Asian	6,147 (3.2%)	799 (2.7%)
American Indian or Alaskan Native	155 (0.1%)	42 (0.1%)
Other/Unknown	71,408 (37.0%)	6,066 (20.2%)

[a] Residents of Harris County Jurisdiction Area. Community cases include all local SARS-CoV-2 infections; [b] Residents of Harris County Jurisdiction area who were hospitalized and tested positive for SARS-CoV-2 infections; [c] White, Black, and Asian include non-Hispanic individuals self-identifying as noted.

Supplemental Table 2. Demographic characteristics of SARS-CoV-2–positive individuals in Harris County and Texas Medical Center by outbreak waves

**May 12, 2020 – September 6, 2020**		
	Community Residents[a] (N=63,026)	Inpatients[b] (N=10,494)
**Age (years)**		
<10	2,063 (3.3%)	607 (5.8%)
10–19	5,574 (8.9%)	488 (4.7%)
20–39	25,646 (40.7%)	1,998 (19.0%)
40–64	23,337 (37.1%)	4,340 (41.4%)
65+	6,349 (10.1%)	3,061 (29.2%)
**Sex**		
Female	32,317 (51.3%)	687 (6.5%)
Male	27,332 (43.4%)	710 (6.8%)
Missing/Unknown	3,377 (5.4%)	9,097 (86.7%)
**Race/Ethnicity[c]**		
Hispanic or Latino	20,016 (31.8%)	4,768 (45.4%)
White	6,508 (10.3%)	1,489 (14.2%)
Black	4,659 (7.4%)	1,877 (17.9%)
Asian	1,062 (1.7%)	214 (2.0%)
American Indian or Alaskan Native	51 (0.1%)	12 (0.1%)
Other/Unknown	30,730 (48.8%)	2,134 (20.3%)
**September 27, 2020 – May 15, 2021**		
	Community Residents[a] (N=120,855)	Inpatients[b] (N=16,931)
**Age (years)**		
**<10**	6,559 (5.4%)	652 (3.9%)
**10–19**	16,837 (13.9%)	648 (3.8%)
**20–39**	42,671 (35.3%)	2,829 (16.7%)
**40–64**	43,087 (35.7%)	6,994 (41.3%)
**65+**	11,650 (9.6%)	5,808 (34.3%)
**Sex**		
**Female**	59,646 (49.4%)	934 (5.5%)
**Male**	51,985 (43.0%)	972 (5.7%)
**Missing/Unknown**	9,224 (7.6%)	15,025 (88.7%)
**Race/Ethnicity[c]**		
**Hispanic or Latin**x	47,072 (38.9%)	6,220 (36.7%)

**White**	19,559 (16.2%)	3,885 (22.9%)
**Black**	11,002 (9.1%)	2,884 (17.0%)
**Asian**	4,780 (4.0%)	533 (3.1%)
**American Indian or Alaskan Native**	98 (0.1%)	24 (0.1%)
**Other/Unknown**	38,344 (31.7%)	3,385 (20.0%)

[a] Residents of Harris County Jurisdiction Area. Community cases include all local SARS-CoV-2 infections; [b] Residents of Harris County Jurisdiction area who were hospitalized and tested positive for SARS-CoV-2 infections; [c] White, Black, and Asian include non-Hispanic individuals self-identifying as noted. Number of Harris County residents with missing age information: 57 (May 12, 2020 – September 6, 2020) and 51 (September 27, 2020 – May 15, 2021).

Supplemental Figure 1. SARS-CoV-2 growth rate, acceleration and stage among Harris County residents (left) and a subgroup of Harris County residents admitted to Texas Medical Center (TMC) hospitals with a positive SARS-CoV-2 test (right) from March 1, 2020 to March 30, 2022

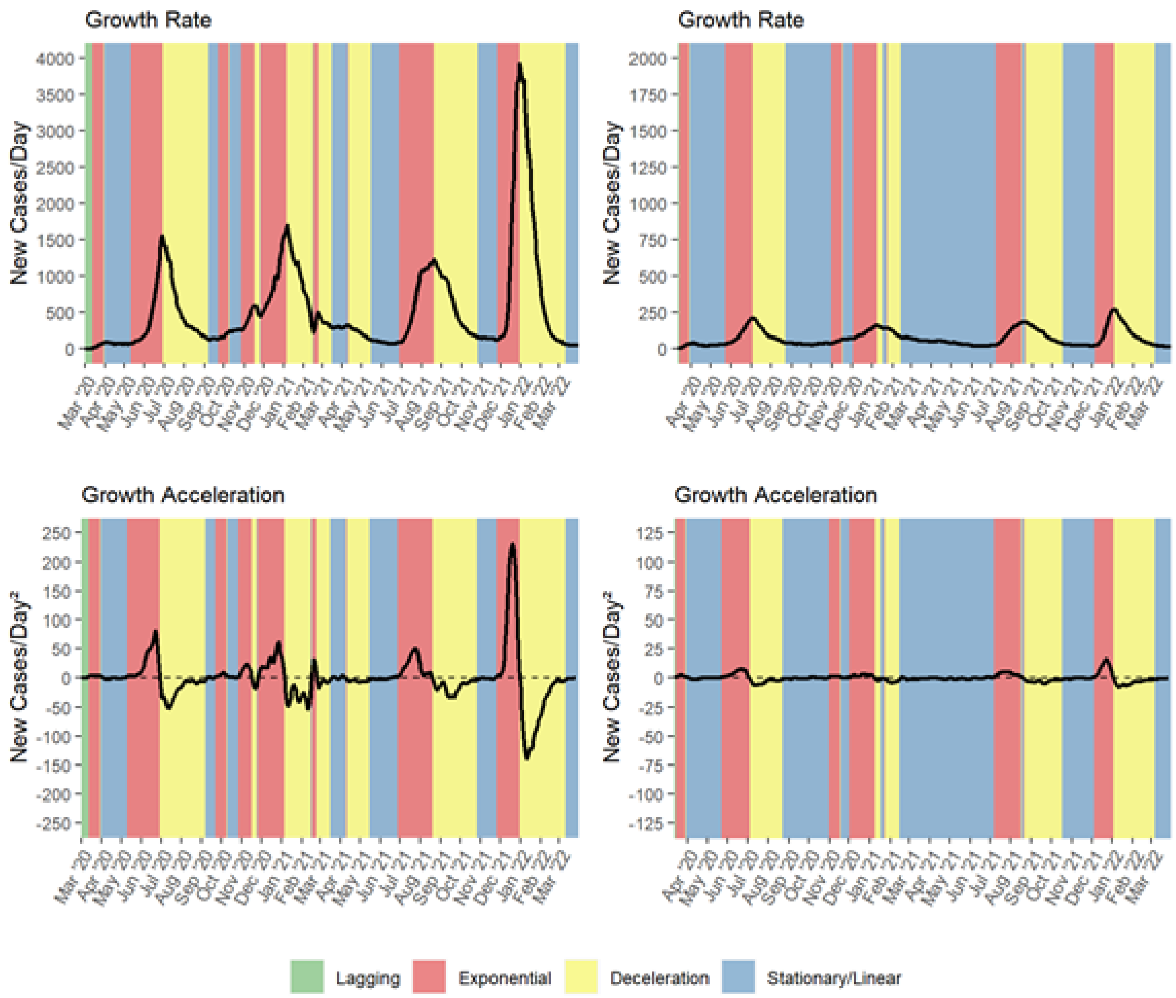

Supplement: Supplementary file 1 — Supplementary Information. [file 41598_2022_20502_MOESM1_ESM.pdf]
